# Spatiotemporal and hotspot detection of U5-children diarrhea in resource-limited areas of Ethiopia

**DOI:** 10.1038/s41598-020-67623-0

**Published:** 2020-07-03

**Authors:** Bezuayehu Alemayehu, Birhanu Teshome Ayele, Claudio Valsangiacomo, Argaw Ambelu

**Affiliations:** 1 Department of Environmental Health Science and Technology, Jimma University, Jimma, Ethiopia; 2 Division of Epidemiology and Biostatistics, Faculty of Medicine and Health Sciences, Stellenbosch University, Stellenbosch, South Africa; 3 University of Applied Sciences and Arts of Southern Switzerland (SUPSI), Manno, Switzerland

**Keywords:** Gastrointestinal diseases, Diseases, Health care

## Abstract

Under-five children (U5-children) diarrhea is a significant public health threat, where the World Health Organisation (WHO) reported it as the second leading cause of children’s death worldwide. Nearly 1.7 billion cases occur annually with varied temporal and spatial factors. Identification of the spatiotemporal pattern and hotspot areas of U5-children diarrhea can assist targeted intervention and provide an early warning for more effective response measures. This study aimed at examining spatiotemporal variability along with the detection of hotspot areas for U5-children diarrhea in the Bench Maji Zone of southwestern Ethiopia, where resources are limited and cultural heterogeneity is highest. Retrospective longitudinal data of ten years of diarrhea records from January 2008 to December 2017 were used to identify hotspot areas. The incidence rate per 1,000 per year among children was calculated along with seasonal patterns of cases. The spatiotemporal analysis was made using SaTScan version 9.4, while spatial autocorrelations and hotspot identification were generated using ArcGIS 10.5 software. A total of 90,716 U5-children diarrhea cases were reported with an annual incidence rate of 36.1 per 1,000 U5-children, indicating a relative risk (RR) of 1.6 and a log-likelihood ratio (LLR) of 1,347.32 (p < 0.001). The highest incidence of diarrhea illness was recorded during the dry season and showed incidence rate increment from October to February. The risky clusters (RR > 1) were in the districts of Bero, Maji, Surma, Minit Shasha, Guraferda, Mizan Aman Town, and Sheko with annual cases of 127.93, 68.5, 65.12, 55.03, 55.67, 54.14 and 44.97 per 1,000, respectively. The lowest annual cases reported were in the four districts of Shay Bench, South Bench, North Bench, and Minit Goldiya, where RR was less than a unit. Six most likely clusters (Bero, Minit Shasha, Surma, Guraferda, South Bench, and Maji) and one lower RR area (North Bench) were hotspot districts. The U5-children's diarrhea in the study area showed an overall increasing trend during the dry seasons with non-random distribution over space and time. The data recorded during ten years and analyzed with the proper statistical tools helped to identify the hotspot areas with risky seasons where diarrhea could increase.

## Background

It is claimed that the global estimates of U5-children's diarrhea are showing a decline. However, it is still a significant cause of morbidity all over the world^1^, especially in developing regions characterized by rapid population growth, increased urbanization, limited infrastructures, and poor healthcare system^2^. Every year, about 2.4 billion of acute diarrhea episodes are occurring across the world^3^. About 10% of all deaths at the global scale is caused by diarrhea, where the problem persists mainly in South Asia and Sub-Saharan African countries^4^. In Ethiopia, the findings from 31 studies revealed 22% of U5-children diarrhea, mostly in the Southern Nations Nationalities and Peoples’ Region(SNNPR), the prevalence was found to be 21%^5,6^.

Ethiopia has been implementing a community health extension program (HEP) since 2003, focusing at the household level, this includes a great effort to reduce the incidence of U5-children diarrhea through the increase of public access to basic health services at the household level. Despite this effort, diarrhea remains a major health problem causing more than 25% of the national morbidity in different parts of the country with a great geographic disparity^7–9^. Among the main reasons, the lack of resources has been identified as a major cause^10^. Despite the presence of several evidences on the predictors of U5-children diarrhea^9,11,12^, there is a limited evidence on spatiotemporal patterns and the detection of hotspot areas in limited-resource settings, such as southwestern Ethiopia. Investigating spatial and temporal patterns of U5-children diarrhea can help to identify the specific hotspot areas with a seasonal pattern for early warning of outbreaks and to take faster mitigation measures^13,14^, spatiotemporal information would be very useful for implementing more efficiently the national public health strategy.

Most of similar studies often failed to address spatial and temporal patterns and were unable to identify significant hotspot areas specifically at the district level. Different analytical tools such as the application of spatial scan statistics, widely used for spatial distribution and space–time cluster analysis of disease surveillance, significantly helps to detect the location and clusters. These tools are also applied to investigate other public health issues, such as respiratory infections, food and water-borne diseases, sexually transmitted diseases, and vector-borne diseases. Clear statistical outputs showing spatial, temporal, and space–time trends are a major advantage of SaTscan compared to other tools. Similarly, geographic information system (GIS) has been currently used for decision support in public health sectors, related to identifying and tracking health-related trends^15,16^.

The evidence on spatiotemporal pattern and hotspot areas of U5-children diarrhea in the Bench Maji Zone is missing or limited for space and time-based interventions. Understanding the role of space and time in spatial and temporal patterns of communicable diseases is critical to a more location-specific and efficient public health intervention to control and prevent under-five children's diarrhea. Besides, analysis of the existing recorded diarrhea data can play a significant role in disease prevention programs in the course of informed decisions that assist control strategies in this study area. Therefore, this study aimed to examine spatiotemporal variability and identify hotspot areas of U5-children diarrhea at the district level in Bench Maji Zone, southwestern Ethiopia.

## Materials and methods

### Study area

**Figure 1:**
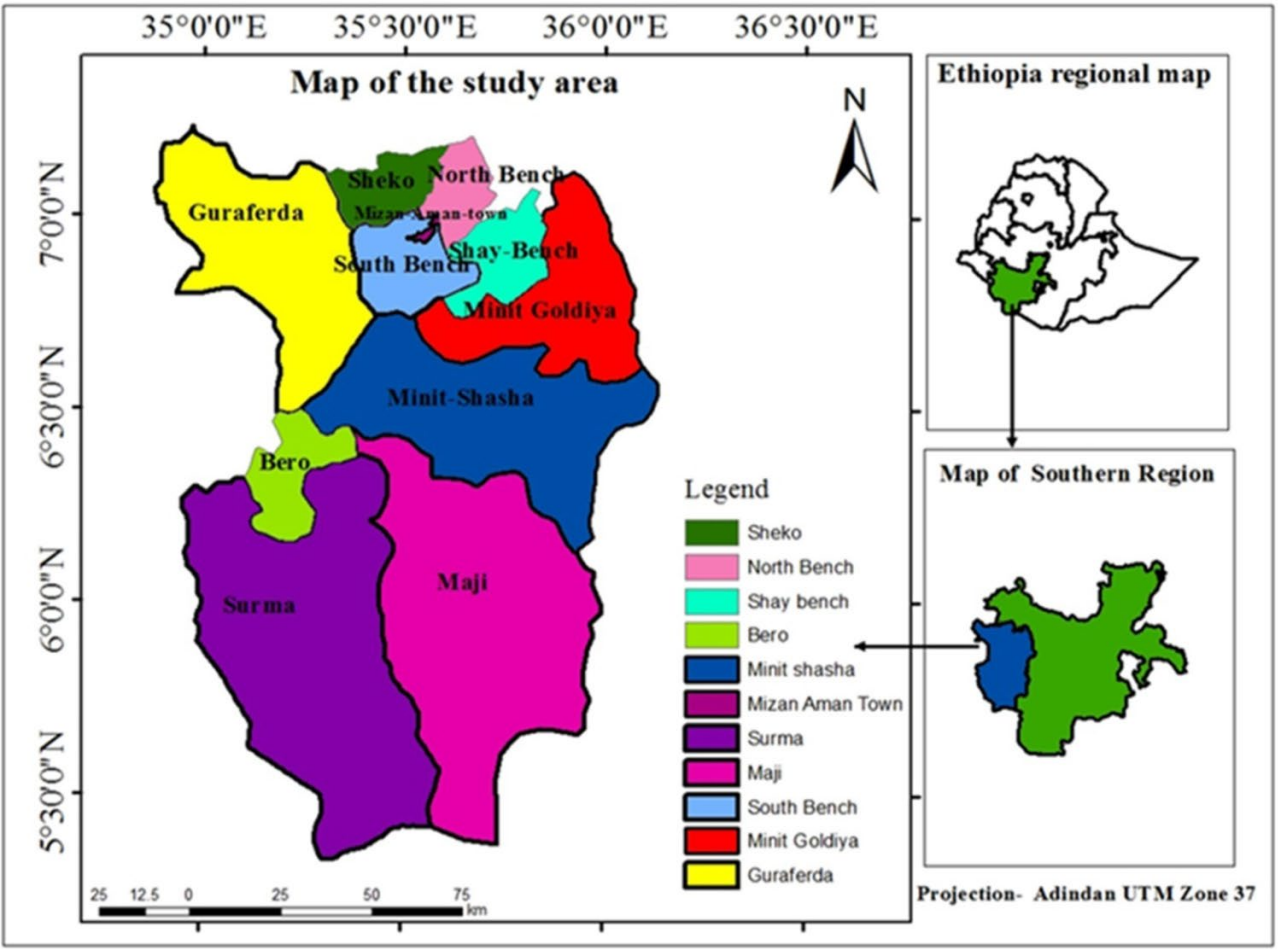
Map of the study area in Bench Maji zone southwest Ethiopia.

The study was conducted in Bench Maji Zone, Southwestern Ethiopia, located in Southern Nations Nationalities and Peoples’ Region (SNNPR) of Ethiopia between 6°27′35.8″ north latitude and 35°18′19.8″ diarrhea, east longitude and 747 m above sea level (Fig. 1). There are eleven districts and one town administration, namely South Bench, Maji, Surma, Bero, Guraferda, North Bench, Sheko, Minit Shasha, Minit Goldiya, Shay Bench, and Mizan Amman Town. The total number of health centers found in the zone was 42 from which the data was retrieved that are maintained in all public health sectors. There are also 382 health extension workers providing basic primary health care services at the household level. The projected population for 2017 was 847,168. Of these, 417,751 were men and 429,417 women^17^. U5-children were estimated to be 196,566. The study area has three rainy seasons, long rainy season (June to September), shorter rainy season (March to May) and the dry season (October to February)^18^. The mean temperature in the study area ranges from 15 to 27 °C, and has total annual rainfall of 400–2008 mm^19^. The previous studies have shown that 20.9% of households use improved sanitation. Almost eighty (79.1%) of households use unimproved sanitation. Less than half (44.6%) of the households use improved drinking water sources. More than half (55.4%) of households use unimproved water sources. Sixty percent (61.3%) of the study area have practice handwashing at critical times, whereas 38.7% do not practice handwashing during critical times^10,20^.

### Study design and period

A retrospective longitudinal study design was used to examine the spatiotemporal variability and detection of hotspot areas of U5-children diarrhea in Bench Maji Zone, southwestern Ethiopia. U5-children diarrhea is identified as passing loose, watery stools three or more times a day in which the onset of the episodes would be less than two weeks among U5-children that offered treatment during visiting health facilities^21^.

### Data collection process

The data on diarrhea among U5-children recorded from 2008 to 2017 were used for analysis. These data were collected from records issued by the Health Management Information System (HMIS), a monthly computer-based data recording and management system used by the Ethiopia Ministry of Health to record, aggregate, analyze, and utilize data to assist health workers, managers and policymakers for evidence-based decisions at all public health facilities^22^. The health data recording system is uniform and maintained in all public health sectors for efficient data management and monthly reporting of cases that are transferred to a central repository in Ethiopia. Health facilities treat and record U5-children diarrhea based on World Health Organization (WHO) guideline that defines diarrhea as passing three or more loose or liquid stools per day, or more frequently than normal and offered the treatment. Missing and consistency of diarrhea data on the recording was checked before analysis^23^. Three data officers were recruited and trained under the supervision of the principal investigator for data collection based on the objectives of the study. The total population of U5-children of each district per year was extrapolated from the result of the 2007 National Population and Housing Census of Ethiopia central statistic agency that is used by the Bench Maji Zone Health Department^24^.

### Organization of the dataset

Monthly-recorded diarrhea cases of U5-children from ten years of HMIS record, total population of U5-children, and types of seasons in the study area were processed using Microsoft Office Excel. Monthly recorded data were compiled to analyze the seasonal distribution of diarrhea, while the average cases of diarrhea were used to identify hotspot areas. Data that is split into two periods were analyzed to determine the pattern of emerging hotspots or long-lasting and yearly diarrhea cases data were used to show the distribution of diarrhea within each district. The compilation of data was implemented using the appropriated analysis method developed to investigate the spatial and temporal pattern of communicable diseases. As the number of study districts was 11 or limited data, the analyses were done based on the number of cases observed during the study period with large datasets^25^.

The coordinate projection was defined by using the World Geodetic System (WGS) 1984, Universal Transverse Mercator (UTM) Zone 37°N. Since the polygons were significantly different in size, the centroids of the polygons were used. The centroids provided information on a specific location and enabled us to undertake the district-level analysis. Subsequently, data were saved in Comma Delimited (CSV) also imported into SaTScan 9.4 software for the spatiotemporal analyses. And we have used ArcGIS 10.5 software to undertake hotspot detection and sketch the map of the study area. The incidence rate per 1,000 per year was calculated in order to discern the distribution of U5-children diarrhea in the study areas, while the incidence rate was log-transformed before performing the seasonal distribution to fulfill the assumptions of normal distribution^25,26^.

### Statistical analysis

#### Spatial scan statistical analysis

SaTscan version 9.4 was used to perform cluster analysis, detect the cluster size, the location, to compute the relative risk, and to test the statistical significance. Monthly diarrhea cases, the number of U5-children population, and the coordinates of the study areas were used as input variables for the discrete Poisson model, with the assumption that cases in each district have a Poisson distribution with a known population of U5-children that are at risk for diarrhea. For maximum spatial size, 50% of the population at risk was used. Purely spatial analysis was adjusted to scan for areas with either high or low rates simultaneously to make the correct statistical inference. The relative risk (RR) of U5-children diarrhea in each district during the study periods was calculated as:

1 $$RR=\frac{c/E[c] }{(C-c)/C-E(c)},$$

where c is the number of observed cases within the cluster, and C is the total number of cases in the dataset.

Note that, since the sample size was small, the analysis is conditioned on the total number of cases observed, E[C] = C. A RR value greater than 1 is used to adjust for an increased risk and a value of less than 1 to adjust for lower risk, whereas a relative risk of zero was used to adjust for missing data for that particular time and location. The population estimates used for the denominators were the average population of U5-children during the decade from 2008 to 2017. Annual rate per 1,000 was calculated, taking leap years into account and is based on the average length of a year^27,28^.

The likelihood ratio was analyzed to measure the relative risk and identify the most likely clusters of the study communities. The maximum likelihood ratio with more observed cases than the expected was identified as the most likely clusters of U5-children. For each location and size of the scanning window, the alternative hypothesis is that there is an elevated risk within the window as compared to outside under the assumptions of Poisson distribution. The likelihood function for a specific window is proportional to:

2 $$\left(\frac{\mathrm{C}}{E\left[c\right]}\right){\left(\frac{\mathrm{C}-\mathrm{c}}{C-E\left[c\right]}\right)}^{C-c}I(),$$

where C is the total number of cases, c is the observed number of cases within the window and E[c] is the expected number of cases within the window under the null-hypothesis. Note that since the analysis was based on the total number of cases observed, C − E[c] is the expected number of cases outside the window. I() is an indicator function. The program was adjusted to scans for clusters with either high or low rates, then I() = 1 for all windows. The expected number of cases in each area under the null hypothesis was calculated using the formula:

3 $$ {\text{E}}\left[ {\text{c}} \right] = {\text{p}}*{\text{C}}/{\text{P}} $$

where c is the observed number of cases and p, U5-children population in each district, while C and P are the total number of cases and population respectively^25^.

#### Space–time scan statistic

The space–time scan statistic was analyzed to identify the highest clustering of U5-children diarrhea corresponding to each district and it’s time for potential clusters. The space–time statistic assumes that the relative risk of the case was the same within the window compared to outside. The Poisson probability model was used for this analysis because U5-children is known to be a population at risk for diarrhea. The level of significance for analysis was confirmed by comparing the likelihood ratio results against a null distribution computed from a Monte Carlo simulation. The number of permutations was set to 999 at P < 0.05 which was considered to be statistically significant^25^.

#### Spatial variation in temporal trends scan statistic

The temporal trend analysis of U5-children diarrhea was calculated inside and outside the scanning window (time period) for each district. The spatial variation with temporal trends provides the estimated time trends inside and outside that detected clusters on the log-linear scale where the annual percentage increases or decreases in the risk. A decreasing trend is characterized by a negative number while increasing trends are associated with non-negative numbers in the table. While doing the spatial and temporal trend analyses, the null hypothesis was ‘trends are the same in among the spatial and temporal clusters’, while the alternative hypothesiswas ‘trends among clusters are different’. The most likely cluster for the temporal trend inside the window is less likely to be the same as the temporal trend outside the cluster. This could happen when the higher cluster exists inside the temporal trend and all areas have the same incidence rate at the beginning of the period, but the cluster area has a higher rate at the end of the period^25^.

#### Spatial autocorrelation analysis

The spatial autocorrelation of U5-children diarrhea from 2008 to 2017 was performed in order to observe whether the pattern expressed is a clustered, dispersed, or random pattern. The Moran’s index, both z-score, and p-value evaluate the significance of that index in which values near + 1.0 indicate clustering while negative, values near − 1.0 indicate dispersion patterns of U5-children diarrhea. The null hypothesis for analysis is no spatial clustering of U5-children diarrhea in the study areas. When the p-value is small and the absolute value of the z- score is large enough that it falls outside the desired confidence level, then the null hypothesis can be rejected. If the index value is greater than 0, U5-children diarrhea exhibits a clustered pattern, whereas if the value is less than 0, it exhibits a dispersed pattern^29^.

#### Detection of hotspot areas of U5-children diarrhea

Getis-Ord Gi statistic was used to identify cases with either high or low values spatially based on z-scores and p-values. Clusters of high values could be hotspot when the z-score is large and positive, whereas cold spot areas could be significantly clustered when there is a small and negative z-scores value. To observe the hotspot variability of U5-children diarrhea, case data were categorized into 2008–2012 and 2013–2017 to identify whether the hotspot area is emerging or long-lasting during the study period. Finally, averages of cases were calculated to identify hotspot districts of U5-children diarrhea, whereas the long-lasting U5-children diarrhea indicates when hotspot areas last for greater than one year in a particular place^30^.

The High/Low clustering analysis results were interpreted within the context of a null hypothesis, i.e. "there is no spatial clustering of U5-children diarrhea". When the absolute value of the z-score is large and the p-value is very small, the null hypothesis can be rejected. The sign of the z-score shall be considered when the null hypothesis is rejected. A positive z-score value indicates that there is high clustering in the study area. The p-value associated with a 95% confidence level is 0.05. If the z-score is between − 1. 96 and + 1.96, the p-value would be larger than 0.05, and could not reject the null hypothesis; the pattern exhibited could very likely be the result of random spatial processes. If the z-score falls outside the range, the observed spatial pattern is probably too unusual to be the result of random chance, and the p-value would be smaller to reflect this^31^.

## Results

**Figure 2:**
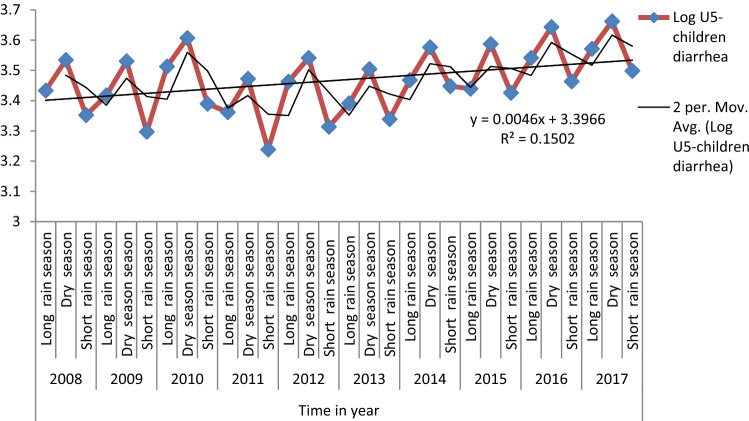
Seasonal distributions of U5-children diarrhea, Bench Maji Zone, southwest Ethiopia, 2008–2017.

Table 1 shows the yearly average case distribution of U5-children diarrhea at the district level attributed to the 90,716 U5-children diarrhea reported during the decade 2008–2017 U5-children. The average case occurrence of diarrhea in 2008 and 2017 was 93.73 and 114.71, respectively. The highest average (160.18) was scored in 2007 in Bero and the lowest average (27.53) was observed in Shay Bench district in 2011. As shown in Fig. 2, the peak incidence rate is recorded during the dry season (from October to February), a slight rise in long rain (from June to September), and the lowest case was observed during the short rain (from March to May) season. There is significant correlation between season and the occurrences of U5-children diarrhea peaking during the dry seasons [Kruskal–Wallis test: H (2, N = 2,640) = 18.66282 p = 0.0001] (Fig. 2). In general, the U5-children diarrhea illness increased during the decade from 2008 to 2017.

**Table 1:** Yearly average case distribution of U5-children diarrhea at the district level, Bench Maji zone, southwest Ethiopia, 2008–2017.

Districts	2008	2009	2010	2011	2012	2013	2014	2015	2016	2017
Bero	113.99	110.89	191.23	156.82	141.81	200.76	161.57	191.02	204.35	160.18
Guraferda	112.27	111.26	125.92	90.83	146.24	102.7	94.1	108.9	91.9	142.48
Maji	144.8	127.28	152.87	116.41	153.13	116.81	148.43	110.89	136.69	169.4
Minit Goldiya	43.3	40.5	51.76	37.67	68.35	24.73	60.87	52.96	64.05	84.31
Minit Shasha	94.92	82.97	105.83	72.92	75.07	110.75	134.43	136.34	162.56	118.57
Mizan Aman	119.64	117.06	144.1	100.48	103.26	73.25	98.89	74.33	101.39	157.88
North Bench	37.87	40.98	45.89	33.12	40.9	54.55	61.03	61.17	62.86	58.7
Shay Bench	37.02	30.88	34.92	27.53	28.5	31.44	44.56	45.3	50.85	54.71
Sheko	85.79	88.26	101.17	63.46	64.26	78.29	92.44	90.14	102.63	140.77
South Bench	45.02	36.19	40.62	30.48	33.96	40.93	53.2	52.31	58.38	67.43
Surma	196.41	173.44	205.87	123.17	152.65	91.72	104.9	82.44	98.02	107.41
Average	93.73	87.25	109.11	77.54	91.65	84.18	95.86	91.44	103.06	114.71

### Spatial distribution of U5-children diarrhea

In Table 2, the overall incidence of diarrhea was 36.120 per 1,000 U5-children population (RR = 1.6, LLR 1,347.32, p < 0.001). The significantly high rate clusters were observed in seven districts, namely Bero, Maji, Surma, Minit Shasha, Guraferda, Mizan Aman town and Sheko, with an annual average per 1,000 of 127.93, 68.55, 65.12, 55.03, 55.67, 54.14 and 44.96, respectively. These seven districts were identified as the most likely clusters with relative risk greater than1 (RR > 1) characterized by rather more cases than expected. The maximum likelihood ratio (LLR+) indicates the significantly high distribution of diarrhea. Low cases were identified in four districts, namely Shay Bench, South Bench, North Bench, and Minit Goldiya, with the RR less than 1, characterized by lower observed cases than expected. Both high and low rate clusters showed significant variation among districts (p < 0.001).

**Table 2:** Spatial clusters of U5-children diarrhea in Bench Maji zone, southwest Ethiopia, 2008–2017.

Name of districts	U5-children population	Observed cases	Expected cases	Annual case/1,000	RR*	LLR+
Bero	47,710	6,105	1,722	127.93	3.73	3,451.37
Shay Bench	44,469	8,645	16,056	19.44	0.49	2,415.49
Maji	11,956	8,197	4,316	68.55	1.99	1,464.53
South Bench	42,058	9,520	15,186	22.63	0.48	1,427.81
North Bench	40,798	10,076	14,730	24.69	0.64	967.91
Surma	9,534	6,209	3,442	65.12	1.86	940.00
Minit Shasha	16,537	9,101	5,971	55.03	1.58	764.31
Guraferda	13,673	7,613	4,937	55.67	1.59	663.28
Mizan Aman	14,074	7,621	5,081	54.14	1.55	587.25
Minit Goldiya	34,021	8,927	12,284	26.24	0.7	578.26
Sheko	19,348	8,702	6,986	44.97	1.27	213.03
Total	251,239	90,716	90,711	36.12	1.6	1,347.32

### Space–time variability of U5-children diarrhea

As shown in Table 3, the highest numbers of annual cases (113.02/1,000) and highest relative risks (R = 3.33) were observed in two districts approximately 50 km apart:Bero and Surma, from 2008/1/1 to 2012/12/31 (LLR = 3,745.13, p < 0.001) respectively.The period from 2008 to 2012 indicating the time in which there is a high clusters of diarrhea occurred in districts of Bero and Surma with the highest relative risk (RR) of diarrhea. Similarly, the annual cases in Maji and Minit Shasha, approximately 14 km apart, were 67.03/1,000 from 2013/1/1 to 2017/12/31. Whereas, the lowest annual cases (18.44/1,000) and lower relative risk (0.44) were observed in Minit Goldiya, Shay Bench and South Bench, approximately 28 km apart, from 2009/1/1 to 2013/12/31. The occurrences of annual cases were significantly diverse in both space and time (p < 0.001).

**Table 3:** Space–time variability of U5-children diarrhea in Bench Maji zone, southwest Ethiopia, 2008–2017.

Name of districts	Coordinates	Time frame	Population	Observed case	Expected case	Annual case/1,000	RR	LLR	p-value
Bero & Surma	5.555278 N, 35.276389 E, 49.51 km	2008/1/1 to 2012/12/31	14,305	7,778	2,484.5	113.0.2	3.33	3,745.1	0.001
Maji & Minit Shasha	6.177500 N, 35.598056 E/13.96 km	2013/1/1 to 2017/12/31	28,493	9,767	5,260.6	67.03	1.96	1658.1	0.001
Guraferda & Mizan Aman	6.865556 N, 35.346111 E/30.54 km	2012/1/1 to 2016/12/31	27,747	7,594	4,696.3	58.37	1.67	801.2	0.001
Minit Goldiya, Shay Bench & South Bench	6.933056 N, 35.876667 E/27.68 km	2009/1/1 to 2013/12/31	120,549	10,853	21,250	18.44	0.44	3,846.6	0.001

### Spatial variation in temporal trends of U5-children diarrhea

Table 4 shows the spatial variation over time (seasons) of U5-children diarrhea. There is a 1.98% overall increase of U5-children diarrhea with spatial and temporal variations (p < 0.001) among the districts. Districts such as South Bench, North Bench, Shay Bench, Minit Goldiya, Minit Shasha, Sheko and Maji were in an increasing pattern in both inside and outside the window, whereas Surma, Bero, MizanAmanTown, and Guraferda districts were in decreasing pattern inside but in an increasing trend in the outside window.

**Table 4:** Spatial variation in temporal trends of U5-children diarrhea in Bench Maji zone, southwest Ethiopia, 2008–2017.

Name of districts	Observed cases	Expected cases	RR	Annual cases/1,000	% of cluster increase or decrease		
					Inside time trend	Outside time trend	LLR
Surma	6,209	3,442.37	1.86	65.12	− 9.55	2.88	392.50
Bero	6,105	1722.77	3.73	127.93	− 8.66	2.81	326.9
South Bench	9,520	15,186.13	0.58	22.63	6.26	1.49	73.13
North Bench	10,076	14,730.97	0.64	24.69	6.02	1.48	70.24
Shay Bench	8,645	16,056.71	0.49	19.44	5.59	1.63	46.97
Minit Goldiya	8,927	12,284.24	0.7	26.24	5.98	1.54	60.51
Minit Shasha	9,101	5,971.03	1.58	55.03	6.13	1.55	65.70
Mizan Aman	7,621	5,081.66	1.55	54.14	− 0.83	2.19	26.04
Guraferda	7,613	4,937.15	1.59	55.67	− 0.37	2.2	18.74
Sheko	8,702	6,986.08	1.27	44.97	3.9	1.78	13.67
Maji	8,197	4,316.89	1.99	68.55	0.32	2.17	10.53
Total	90,716	90,716	1.45	36.10	1.34	1.98	100.45

### Spatial autocorrelation patterns of U5-children diarrhea

Table 5 shows the spatial autocorrelation patterns of U5-children diarrhea. The Global Moran’s index values in all years except in 2008 and 2012 were higher than zero, and in which positive values indicate that U5-children diarrhea cases exhibited a clustered pattern at the district level every year. The negative values for the index for the years 2008 and 2012 indicated outlier with dissimilar clusters and representing low clustering times of U5-children diarrhea.

**Table 5:** Spatial autocorrelation patterns of U5-children diarrhea in Bench Maji zone, southwest Ethiopia, 2008–2017.

Year	Moran's index	Expected index	Variance	z-score	p-value	Pattern
2008	− 0.22	− 0.1	0.02	− 0.81	0.42	Clustered
2009	0.12	− 0.1	0.03	1.48	0.14	Clustered
2010	0.17	− 0.1	0.02	− 0.5	0.62	Clustered
2011	0.32	− 0.1	0.02	− 1.49	0.14	Clustered
2012	− 0.13	− 0.1	0.02	− 0.18	0.86	Clustered
2013	0.16	− 0.1	0.02	− 0.4	0.69	Clustered
2014	0.17	− 0.1	0.02	1.81	0.07	Clustered
2015	0.1	− 0.1	0.02	1.24	0.21	Clustered
2016	0.2	− 0.1	0.02	2.02	0.04	Clustered
2017	0.39	− 0.1	0.02	3.32	< 0.001	Clustered

### Identifications of hotspot districts of U5-children diarrhea

**Figure 3:**
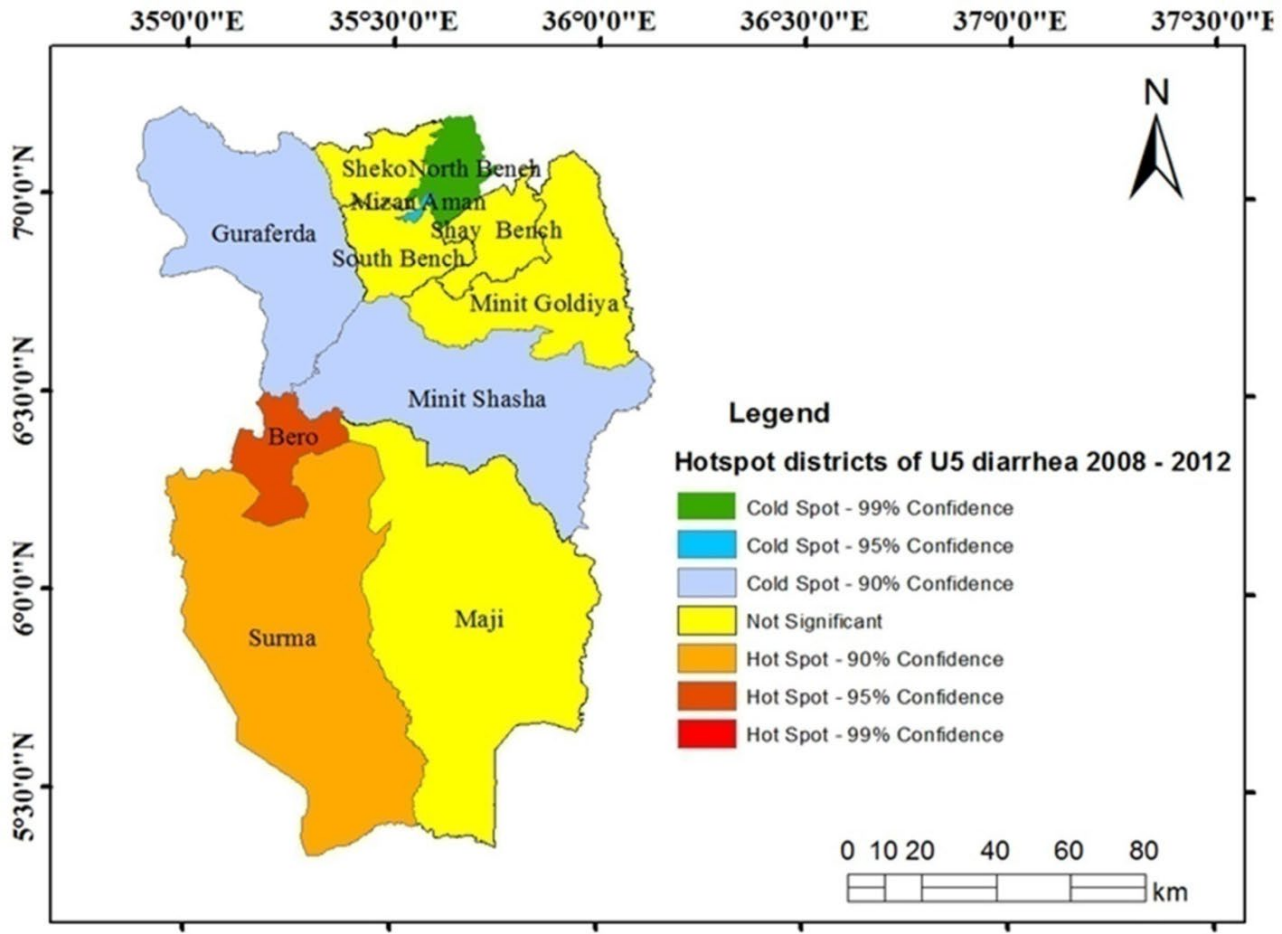
Hotspots districts of U5-children diarrhea, Bench Maji zone, southwest Ethiopia, 2008–2012.

The hotspot variability of U5-children diarrhea was detected at different times. In Fig. 3, two hotspot areas were identified between 2008 and 2012 in Bero and Surma.

**Figure 4:**
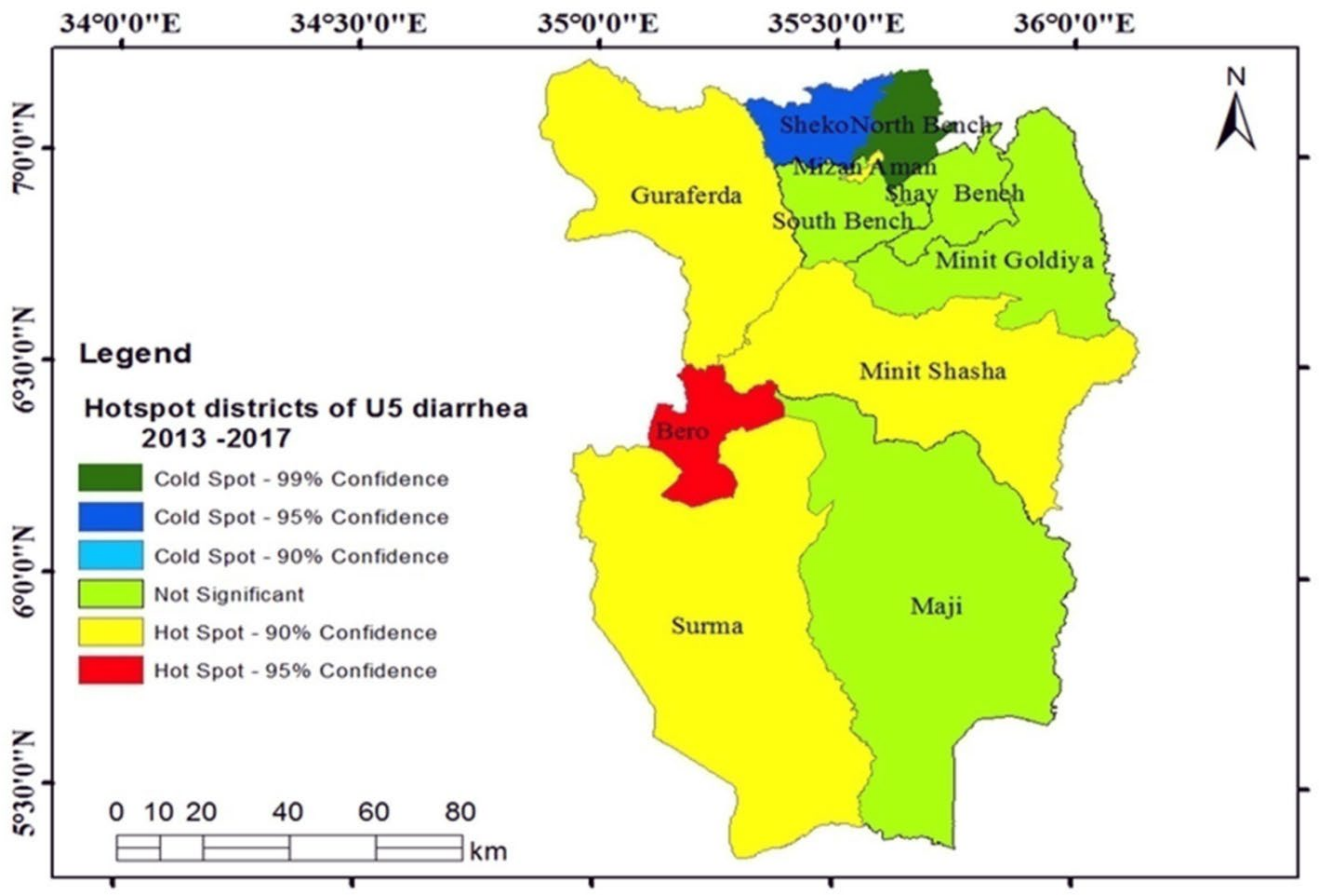
Hotspots districts of U5-children diarrhea, Bench Maji zone, southwest Ethiopia, 2013–2017.

Likewise, hotspot areas detected during 2013–2017 were Bero, Minit Shasha, Surma, Guraferda, Minit and Mizan Aman Town (Fig. 4).

**Figure 5:**
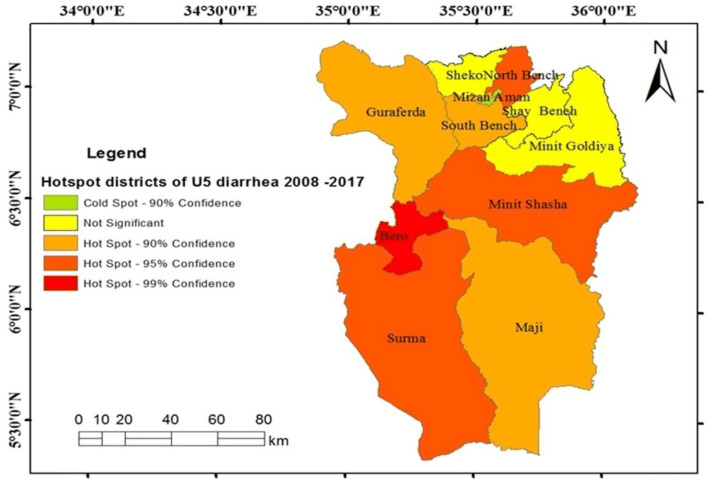
Hotspots districts of U5-children diarrhea in Bench Maji zone, southwest Ethiopia, 2008–2017.

In Fig. 5, the overall hotspot map of the study region for the ten years (2008–2017) indicated, Bero, North Bench, Minit Shasha, Surma, Guraferda, South Bench, and Maji to be hotspot areas. However, the analysis indicated that Minit Goldiya, Sheko, Shay Bench, and Mizan Aman Town had no significantly classified as hotspot areas for the ten years of period.

## Discussion

This study revealed that the spatial distributions of U5-children diarrhea were clustered and showed the presence of hotspot areas. This information is particularly useful for prioritizing intervention areas by public health officials operating within the community health extension program (HEP) promoted by the Ethiopian government. The most significant spatial cluster was detected in seven districts [Bero, Maji, Surma, Minit Shasha, Guraferda, Mizan Aman town, and Sheko (RR > 1)], whereas four districts (Shay Bench, South Bench, North Bench and Minit Goldiya) were identified as lower rate with lower relative risk. The probable reasons for the spatial variability of diarrhea distribution among the districts might be previously identified risk factors such as untreated drinking water, open field defecation, lack of handwashing facilities and low utilization of primary healthcare services at the household-level^32^.

This study shows that U5-children diarrhea is a 1.98% overall increasing pattern with significant temporal and spatial variation among the districts. The difference in the spatial and temporal pattern of U5-children diarrhea might be due to the geographic disparities^33^ or heterogeneity/inequality in socio-economic activities in which the study areas were characterized by agro-pastoral communities with inadequate health services^12^. An increasing trend of U5-children diarrhea in this study was similar to the findings from Sidama Zone, Southern Ethiopia^34^. However, it is inconsistence with the evidence from northwest Ethiopia and Ethiopia Demographic and Health survey (EDHS) reports in 2016. The variability in trends might be due to proper implementations of health extensions program at the household level and initiations of the rotavirus vaccination program during the study period. This program was introduced in the Ethiopia Expanded Program on Immunization (EPI) in November 2013. Whereas the dissimilarity in trends might be due to the differences in study designs in which EDHS used a cross-sectional household survey targeting children who had diarrhea 2 weeks before the survey at the country level. In contrast, this study used a retrospective approach based on children who had diarrhea, visited public health facilities and received treatment^17^. Moreover, resource limitation, in both availability and program utilization, specifically, incomplete implementation of primary health care units, particularly low coverage of rotavirus vaccine and less exclusive breastfeeding during the first 6 months of life as a key child survival intervention^35^ may have contributed to the case burden.

This study identified a seasonal distribution pattern of U5-children diarrhea, showing an increase of cases during the dry season from October to February and a variation across the season. This is similar to the increasing trends in geographically remote areas of Ethiopia^36^ that might be due to the shortage of safe and adequate drinking water supply^37^, the shortage of rainfall during the dry season which was related to an increased prevalence of U5-children diarrhea^38^. A study have shown that the maximum temperature was positively associated with the increased exposure to bacteria and shortage of drinking water that may affect U5-children^39,40^. Further, the sentinel surveillance from other areas in Ethiopia identified rotavirus as a pathogen responsible for U5-children diarrhea^41,42^.

An increasing pattern of clusters in this study was consistent with findings of Ghana from 2010 to 2014, but inconsistent with Uganda and Mozambique cases, were prevalence was observed during rainy seasons. In fact, heavy rainfalls may contaminate drinking water facilities through surface wash out of contaminated soils and wash out and/or disruption of sanitation services^43,44^. The similarities in an increasing trend of U5-children diarrhea for longer times indicate the recurrent transmission of diarrhea in this specific study area^45^. Moreover, this study identified clustering patterns of U5-children diarrhea throughout the study period, implying that the risk factors have not been changed over the time^46^. This study identified further the non-random distribution and clustered patterns of U5-children diarrhea which is consistent with the previous study analyzed by using EDHS data in Ethiopia^36^.

This study identified hotspot areas of U5-children diarrhea which is similar to the studies done in northwest Ethiopia, Thailand and Ghana that was used to prioritize early prevention strategies of diarrhea^34,47^. Moreover, our study showed that the areas with the highest relative risks such as Bero, North Bench, Minit Shasha, Surma, Guraferda, South Bench, and Maji were identified as a hotspot, but Mizan AmanTown with the highest relative was not exhibited as a hotspot. Similarly, lower incidence rate and lower relative risk of U5-children diarrhea North Bench district) exhibited hotspot. The reason for the inconsistency of the highest relative risks and not exhibiting hotspot may be due to statistical inference, indicating the significance of testing and inference in cluster analysis^48^.

The hotspot variability in space and time was detected in our study. Some areas such as Bero, Surma, Guraferda and Minit Shasha were identified as hotspots at different times (during 2008–2012, 2013–2017 and from 2008 to 2017), exhibiting the persistence and long-term hotspot of diarrhea. This was showing the priority areas for targeted interventions by public health authorities, such as the primary healthcare (Ethiopian community health extension program) at household and community-levels. Moreover, this study area is characterized by pastoral (Surma), semi-pastoral (Bero, and Minit Shasha) and agricultural (Guraferda) communities with shortages of different health services and under the influences of diarrhea since a long time.

The limitations of this study can be summarized as follows: (1) it was not community-based and hence did not analyze the data based on agroecology, urban and rural settings, and demographic variability like gender; (2) there might be missed or causing under estimation of cases when children who did not visit public health facilities for treatment; (3) the potential risk factors which contributed to U5-children diarrhea were not investigated.

In spite of these limitations, the strength of the study could be the use of monthly-recorded data for a longer study period that enabled to examine the spatial, temporal, space–time and identified hotspot areas. This data analysis could contribute to prioritizing interventions by policymakers, health managers and healthcare providers at different levels. Particularly, an increasing trend and the hotspot map provided significant information or evidence to assist health sectors during health resource allocations such as the deployment of health professionals, budget and provisions of infrastructures and strengthening the disease surveillance system. Further, this study can also be a base for hypothesis generation for future research.

## Conclusions

The identification of hotspot areas and temporal incidence of diarrheas (dry season) represents a valuable information for health professionals working within HEP in Ethiopia in order to better implement measures for reducing the burden of this important disease. The investigation generates information that allows to save valuable resources within the public health system, better targeting the hotspot areas at the right time.

The study also detected U5-children diarrhea hotspot areas. The findings of this study provided evidence for public health planners/operators and policymakers about hotspot areas of U5-children diarrhea, allowing for effective interventions and monitoring prevention activities. The increasing of U5-children diarrhea in this study area is suggesting the priority for policy attention at identified hotspot areas focusing on targeted interventional activities such as water, sanitation and provisions of vaccination services.

Generally, this study provided valuable information about the spatiotemporal variability and the hotspot areas of under-five children's diarrhea. This evidence could help to implement targeted interventions and the basis for hypothesis generation to track further study on contributing factors such as the impact of climate change on the occurrence of diarrhea, supplementary feeding status of children and water, hygiene and sanitation status based on agroecology of the areas.

## Data Availability

The datasets used and/or analyzed during the current study are available from the corresponding author on reasonable request.
